# Medial prefrontal theta phase coupling during spatial memory retrieval

**DOI:** 10.1002/hipo.22255

**Published:** 2014-02-18

**Authors:** Raphael Kaplan, Daniel Bush, Mathilde Bonnefond, Peter A Bandettini, Gareth R Barnes, Christian F Doeller, Neil Burgess

**Affiliations:** 1 NIMH-UCL Joint Neuroscience Graduate Partnership Program, National Institute of Mental HealthBethesda, Maryland; 2 Section on Functional Imaging Methods, Laboratory of Brain and Cognition, National Institute of Mental HealthBethesda, Maryland; 3 University College London, Institute of Cognitive NeuroscienceAlexandra House, London, WC1N 3AR, United Kingdom; 4 University College London, Institute of NeurologyLondon, WC1N 1PJ, United Kingdom; 5 Donders Institute for Brain, Cognition and Behaviour, Radboud University Nijmegen6500 HB, Nijmegen, The Netherlands

**Keywords:** oscillations, mPFC, MTL, hippocampus, MEG

## Abstract

Memory retrieval is believed to involve a disparate network of areas, including medial prefrontal and medial temporal cortices, but the mechanisms underlying their coordination remain elusive. One suggestion is that oscillatory coherence mediates inter-regional communication, implicating theta phase and theta-gamma phase-amplitude coupling in mnemonic function across species. To examine this hypothesis, we used non-invasive whole-head magnetoencephalography (MEG) as participants retrieved the location of objects encountered within a virtual environment. We demonstrate that, when participants are cued with the image of an object whose location they must subsequently navigate to, there is a significant increase in 4–8 Hz theta power in medial prefrontal cortex (mPFC), and the phase of this oscillation is coupled both with ongoing theta phase in the medial temporal lobe (MTL) and perceptually induced 65–85 Hz gamma amplitude in medial parietal cortex. These results suggest that theta phase coupling between mPFC and MTL and theta-gamma phase-amplitude coupling between mPFC and neocortical regions may play a role in human spatial memory retrieval. © 2014 The Authors. Hippocampus Published by Wiley Periodicals, Inc.

## INTRODUCTION

Memory retrieval is thought to depend on the coordination of multiple brain regions, and recent theories posit that communication between the hippocampus and neocortex, particularly the medial prefrontal cortex (mPFC), plays a critical role in long-term memory function (e.g., Frankland and Bontempi, 2005; Wang and Morris, 2010; Battaglia et al., 2011; Hyman et al., 2011). One hypothesized mechanism of information exchange between regions is oscillatory coupling (e.g., Fries, 2005; Fell and Axmacher, 2011). In rodents, 6–10 Hz theta oscillations are prominent in the hippocampal formation during spatial exploration (Vanderwolf, 1969; O'Keefe and Nadel, 1978) and theta rhythmicity is associated with mnemonic function (Winson, 1978). In humans, 3–8 Hz theta power in both the medial temporal lobe (MTL; Cornwell et al., 2008; Guderian et al., 2009; Fell et al., 2011; Kaplan et al., 2012; Lega et al., 2012; Staudigl and Hanslmayr, 2013; see Duzel et al., 2010 for review) and frontal midline (Klimesch et al., 2001; Raghavachari et al., 2001; Summerfield and Mangels, 2005; Griesmayr et al., 2010; Addante et al., 2011; see Mitchell et al., 2008 for review) have been correlated with mnemonic function.

Interestingly, rodent studies have demonstrated phase locking of single unit activity in mPFC to ongoing hippocampal theta oscillations during active exploration (Hyman et al., 2005; Siapas et al., 2005); and enhanced phase coupling between the two regions during spatial decision-making (Jones and Wilson, 2005; Young and McNaughton, 2009; Benchenane et al., 2010). Moreover, increases in frontal midline theta power have been demonstrated to co-occur and phase couple with hippocampal theta in freely behaving rodents during exploratory behavior and rearing (Young and McNaughton, 2009). Similarly, phase locking of single unit activity to 3–8 Hz theta oscillations in the human MTL during encoding predicts subsequent memory performance (Rutishauser et al., 2010) and low-frequency (1–10 Hz) phase-locking between mPFC and multiple regions, including the MTL, correlates with successful mnemonic function (Sauseng et al., 2004; Anderson et al., 2010; Watrous et al., 2013).

In a parallel line of research, it has been demonstrated that rodent hippocampal theta occurs alongside 40–100 Hz gamma oscillations during active navigation (Bragin et al., 1995; Chrobak and Buzsaki, 1998). In humans, increases in 30–80 Hz gamma power across occipital regions have been associated with visual perception (Gray and Singer, 1989; Tallon-Baudry and Bertrand, 1999; Hall et al., 2005; Hoogenboom et al., 2006). Moreover, the amplitude of local gamma rhythmicity can be modulated by theta phase in the rodent hippocampal formation during active navigation (Bragin et al., 1995; Chrobak and Buzsaki, 1998; Colgin et al., 2009; Tort et al., 2009; Belluscio et al., 2012), and in the human MTL (Mormann et al., 2005; Axmacher et al., 2010; Staudigl and Hanslmayr, 2013) and neocortex (Canolty et al., 2006; Sauseng et al., 2009; Maris et al., 2011; van der Meij et al., 2012) during mnemonic function. Long-range coupling between hippocampal theta phase and neocortical gamma amplitude has also been identified during spatial exploration in rodents (Sirota et al., 2008). This raises the possibility that theta phase may modulate gamma amplitude in multiple task-relevant regions during cognition (Jensen and Lisman, 1996; for reviews see Lisman and Buzsaki, 2008; Schroeder and Lakatos, 2009; Canolty and Knight, 2010; Jutras and Buffalo, 2010; Fell and Axmacher, 2011).

To ascertain how changes in oscillatory power and coupling might interact during human spatial memory retrieval, we used MEG to measure theta and gamma power across the whole brain during the cued retrieval of object locations within virtual environments and subsequently examined inter-regional theta phase and theta-gamma phase-amplitude coupling during this time period, compared to a baseline period of quiet fixation.

## MATERIALS AND METHODS

### Participants

Seventeen right-handed male participants gave written consent and were compensated for performing the experimental task, as approved by the local research ethics committee at University College London in accordance with Declaration of Helsinki protocols. All participants had normal or corrected-to-normal vision and reported to be in good health with no prior history of neurological disease. Further information about the participants, stimuli, and task can be found in Kaplan et al. (2012), where the same data were analyzed for changes in spectral power related to performance.

### Procedure

**FIGURE 1:**
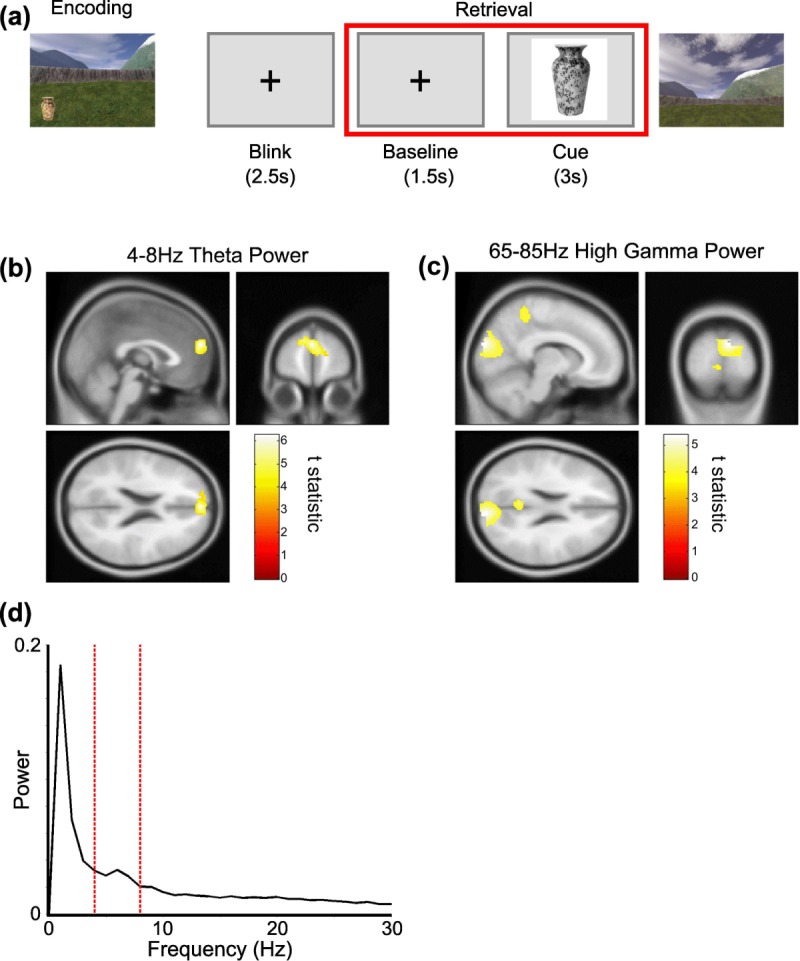
Task structure and oscillatory power changes. (a) Structure of the experiment. Participants first navigate freely in a virtual environment and encode object locations during a learning period. After a short break their memory for these object locations is tested. During these retrieval trials a 1.5-s baseline fixation period is followed by a 3-s cue period (highlighted by the red box), where an image of one object is presented on screen. Participants are subsequently required to navigate to the remembered location of that object in the environment and make a response. (b) A 4–8 Hz theta power source reconstruction group-averaged image showing a significant increase in the mPFC (peak at [0; 58; 22], *Z*-score: 4.39) between baseline and cue periods; and (c) 65–85 Hz high gamma power source reconstruction group-averaged image showing significant increases in the visual cortex (peak at [10; −92; 24], *Z*-score: 4.00) and precuneus (peak at [−4; −50; 34], *Z*-score: 3.80) between cue and baseline periods. Both images shown at the statistical threshold of *P* < 0.001 uncorrected and overlaid on the canonical Montreal Neurological Institute 152 T1 image. (d) Normalized power spectra from a virtual electrode placed at the location in mPFC of the group average peak theta power increase—i.e. [0; 58; 22]—for the cue periods, averaged across *n* = 17 participants. Note the peak in the 4–8 Hz theta band (delimited with dashed red lines). [Color figure can be viewed in the online issue, which is available at http://wileyonlinelibrary.com.]

Participants navigated freely in a virtual environment projected onto a screen in front of them, using a button box with their right hand to move the virtual viewpoint (Kaplan et al., 2012). In each of six sessions, participants were asked to collect and encode the location of six different objects in the environment, three times each (three objects in each of two practice sessions). Object location was constant within each session. Having completed this exploration phase, participants' memory for the object locations was then tested in retrieval trials. In each of six retrieval trials, one per object, a 4-s inter trial interval (ITI) composed of blink phase and baseline fixation period was followed by a 3-s cue period during which an image of one object was presented on screen. Participants were subsequently placed back in the virtual environment and asked to navigate to the remembered location of that object and make a response (Fig. 1a). This provided a total of 36 baseline fixation and cue periods per participant for the analyses presented here.

### MEG Acquisition

Recordings were made using a 275-channel Canadian Thin Films (CTF) MEG system with superconducting quantum interference device (SQUID)-based axial gradiometers (VSM MedTech) and second-order gradients in a magnetically shielded room. Neuromagnetic signals were digitized continuously at a sampling rate of 480 Hz and then band-pass filtered in the 0.1–120 Hz range. Head positioning coils were attached to nasion, left, and right auricular sites to provide anatomical coregistration.

### MEG Source Reconstruction

The linearly constrained minimum variance (LCMV) scalar beamformer spatial filter algorithm from SPM8 (Wellcome Trust Centre for Neuroimaging, London; Litvak et al., 2011) was used to generate source activity maps in a 10-mm grid (Barnes et al., 2003). Coregistration to MNI coordinates was based on nasion, left and right preauricular fiducial points. The forward model was derived from a single-shell model (Nolte, 2003) fit to the inner skull surface of the inverse normalized SPM template. The beamformer source reconstruction algorithm consists of two stages: first, based on the data covariance and lead field structure, weights are calculated which linearly map sensor data to each source location; and second, a summary statistic based on the mean oscillatory power between experimental conditions is calculated for each voxel.

For the analyses presented here, the final 1s baseline fixation period from the 4-s intertrial interval prior to cue onset was contrasted with the period from 0.5 to 1.5 s after cue presentation (corresponding to the period of performance-related hippocampal theta power differences observed in previous analyses of these data; Kaplan et al., 2012) across each of the 36 retrieval trials performed by each participant (see Fig. 1a); and the summary statistic computed for changes in 4–8 Hz theta, 30–45 Hz low- or 65–85 Hz high- gamma power normalized by the projected sensor white noise power and averaged across trials. The choice of low (30–45 Hz) and high (65–85 Hz) gamma frequency bands was primarily motivated by previous studies of phase-amplitude coupling in the rodent medial temporal lobe, which have demonstrated the existence of two distinct (low and high) gamma bands (Bragin et al., 1995; Colgin et al., 2009); and by the observation that the frequency of gamma band oscillations are conserved across mammalian species (Buzsaki et al., 2013).

### Phase Coupling

Instantaneous theta phase in voxel *n* at time *t*, Ø(*t,n*), was extracted from the analytic signal obtained by applying the Hilbert transform to the 4–8 Hz band-pass filtered time series generated by the LCMV beamformer algorithm. The mPFC seed voxel for each participant was chosen as that with the greatest theta power increase between baseline and cue periods within 20 mm of the group maximum co-ordinates, in order to account for the observed variance in frontal midline theta source location between participants (Ishihara et al., 1981; Mitchell et al., 2008). Two metrics were then used to assay theta phase coupling between a single seed voxel and every other voxel in the brain: the phase locking value (PLV) and phase lag index (PLI) (Lachaux et al., 1999; Stam et al., 2007). The PLV is computed as the resultant vector length of phase differences over time, such that a larger value indicates less variability in the phase difference between two signals [Eq. (1)]. The PLI is computed by assigning a value of +1 or −1 at each time step according to whether the phase difference between seed and source voxels is positive or negative and then taking the absolute value of the mean over time, which will tend to zero for randomly distributed phase differences and to one for a consistent nonzero phase relationship [Eq. (2)]. The PLI measure is also designed to ameliorate volume conduction effects by being increasingly less sensitive to coupling effects as phase differences approach zero (Stam et al., 2007). PLV and PLI values for each trial are then averaged for each condition before being entered into a second level statistical analysis.


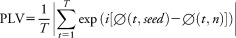


Equation 1: The phase locking value (PLV)


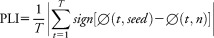


Equation 2: The phase lag index (PLI)

### Phase-Amplitude Coupling

To investigate possible theta phase modulation of gamma amplitude, we examined modified PLV values between a single theta phase seed and the phase of gamma amplitude in all other voxels across the brain within each trial (Vanhatalo et al. 2004; Penny et al. 2008). Instantaneous gamma amplitude in each voxel was obtained by applying the Hilbert transform to the band-pass filtered time series obtained using the LCMV beamformer algorithm. This gamma envelope was then band-pass filtered in the 4–8 Hz theta range, the instantaneous phase of that envelope obtained by applying the Hilbert transform for a second time, and PLV of this phase difference calculated for all voxels in the brain.

### Statistical Analysis

For each of the analyses described above, summary images for each participant were entered into a second level one-sample *t* test in SPM8 with a significance threshold of *P* < 0.001 uncorrected (cluster extent of at least 10 voxels) and *P* < 0.05 family-wise error (FWE) corrected at the cluster-level for multiple comparisons across the whole brain volume. All image coordinates are listed in Montreal Neurological Institute (MNI) space. All images are displayed at the *P* < 0.001 uncorrected threshold, for illustrative purposes, but all effects reported are significant at the *P* < 0.05 FWE cluster-level corrected threshold.

### Additional Control Analyses

Because of the proximity of frontal midline regions to the eyes, we wished to control for any possible influence of eye movements on our measures of changes in oscillatory power between conditions. We therefore obtained time series for eye movements in each participant by applying independent component analysis (ICA) to sensor level MEG data (using EEGlab, Delorme et al., 2004; and the FieldTrip software package, Oostenveld et al., 2011) and isolating eye movement signals based on their appearance and topology. The variance of these eye movement time series was computed for each of the 36 baseline and cue trials, and variance values for all 72 trials were normalized to a mean value of zero and standard deviation of one. The influence of these normalized eye movement variance values on theta and gamma power measurements at every voxel in the brain were estimated by linear regression. This left “residual” theta and gamma power measurements for all 72 trials, whose variance could not be accounted for by changes in eye movements between trials, and these residual values were entered as summary images for each participant into a second-level one sample *t* test in SPM8 with a significance threshold of *P* < 0.001 uncorrected and a cluster extent of at least 10 voxels. Power changes found in this control analysis were assumed to be independent of any linear effects of eye movements. An alternative approach would be to use ICA to remove eye movement components from the sensor level MEG data prior to further analyses (cf., Fatima et al., 2013).

In addition to eye movements, phase coupling measures can be biased by concurrent changes in oscillatory power due to changes in the signal to noise ratio (Muthukumarawamy and Singh, 2011). Hence, to control for any possible influence of changes in oscillatory power or changes in eye movements on our phase and phase-amplitude coupling measures, we repeated the control analysis described above, with additional regressors corresponding to oscillatory power in seed and source voxels for each trial. Specifically, we obtained theta power measurements for the mPFC source voxel in each participant, as well as theta or gamma power measurements for every other voxel in the brain, and normalized the mean and standard deviation of these values to one and zero, respectively, as described above. The influence of these normalized measures of oscillatory power in seed and source voxels, as well as normalized eye movement variance values, on each measure of phase and phase-amplitude coupling were then removed by linear regression. This left “residual” phase and phase-amplitude coupling measures for all 72 trials whose variance could not be accounted for by changes in oscillatory power in the seed or source voxels, or by changes in eye movements, between trials. These residual values were again entered into a second-level one sample *t* test in SPM8 with a significance threshold of *P* < 0.001 uncorrected and a cluster extent of at least 10 voxels. Phase coupling changes found in this control analysis were assumed to be independent of any linear effects of eye movements or of power changes in the source and seed locations.

## RESULTS

### Theta Power Changes and Source Reconstruction

We utilized the linearly constrained minimum variance (LCMV) beamformer algorithm (Barnes et al., 2003) in SPM8 (Litvak et al., 2011) to estimate cortical sources that exhibited significant increases in theta (4–8 Hz), low (30–45 Hz) and high (65–85 Hz) gamma power between baseline periods of quiet fixation and cue periods of putative spatial memory retrieval. We identified a single region in mPFC that exhibited a significant theta power increase during the cue period (peak at [0; 58; 22], *Z* score = 4.39, Fig. 1b). No regions exhibiting significant increases in low gamma power were identified. In the high gamma band, we identified a single region with three distinct peaks in the visual cortex (first peak at [10; −92; 24], *Z*-score = 4.00; second peak at [−8; −86; 36], *Z*-score = 3.79; Fig. 1c) and precuneus (peak at [−4; −50; 34], *Z*-score = 3.80) that exhibited a significant increase in oscillatory power, presumably due to the visual response induced by the presentation of the cue (Gray and Singer, 1989; Tallon-Baudry and Bertrand, 1999; Hall et al., 2005; Hoogenboom et al., 2006). Additional control analyses indicate that these increases in oscillatory power were not due to differences in eye movements between conditions (see Materials and Methods).

### Theta Phase Coupling

Next, we used the mPFC region that exhibited a significant theta power increase between baseline and cue periods as a seed region to investigate changes in theta phase coupling across the whole brain. The specific seed voxel for each participant was chosen as that with the greatest theta power increase between baseline and cue periods within 20 mm of the group maximum, in order to account for the variance in frontal midline theta source locations between participants (Ishihara et al., 1981).

**FIGURE 2:**
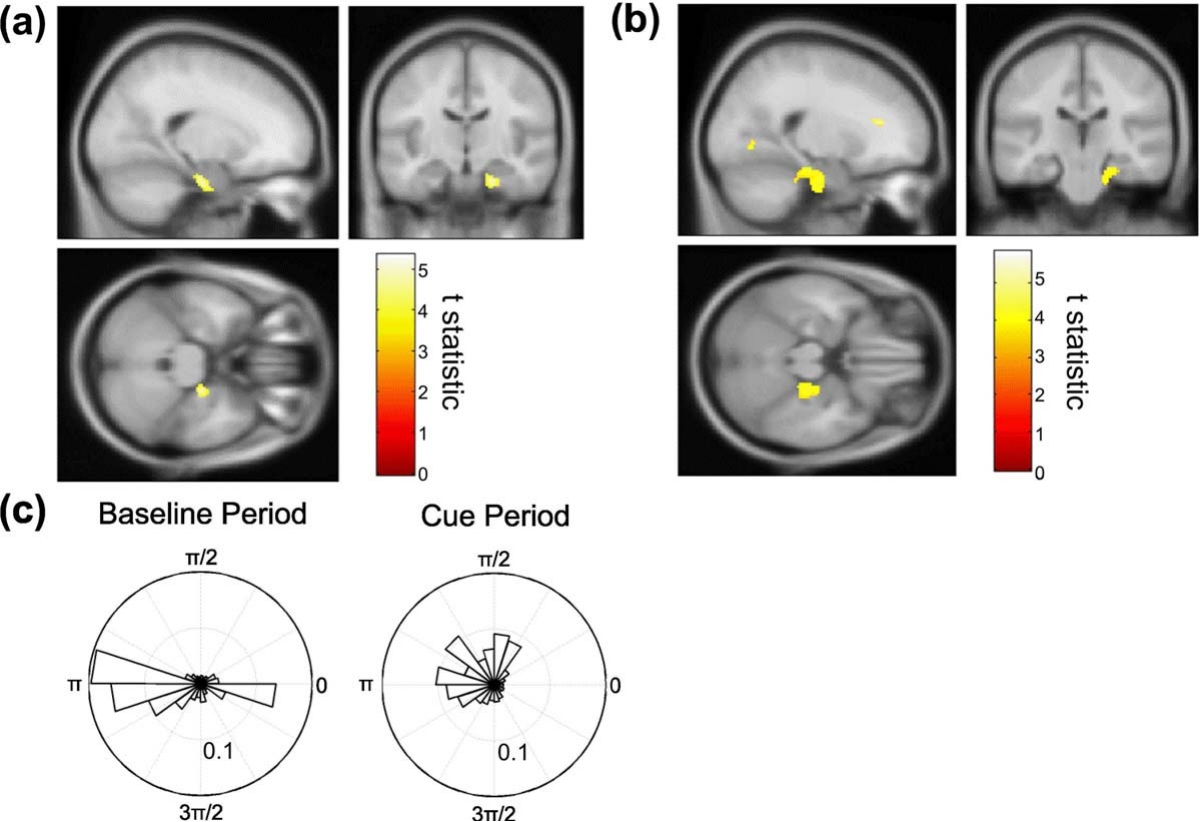
mPFC-aMTL theta coupling during retrieval. Group-averaged brain images showing a significant increase in theta phase coupling between the mPFC seed and right aMTL between baseline and cue periods, identified using the (a) phase locking value (PLV, peak at [20; −16; −26], *Z*-score = 4.52) and (b) phase lag index (PLI, peak at [22; −24; −22], *Z*-score = 3.48). Both images shown at the statistical threshold of *P* < 0.001 uncorrected overlaid on the canonical Montreal Neurological Institute 152 T1 image. (c) Circular histogram of theta phase differences between mPFC seed and aMTL voxel exhibiting the group peak PLV increase for a single subject at each time point during a typical baseline and cue period (in this case the PLI values were: baseline PLV = 0.29, cue PLV = 0.36). Note the narrower distribution of phase differences during the cue period. [Color figure can be viewed in the online issue, which is available at http://wileyonlinelibrary.com.]

First, we used the phase locking value (PLV; see Materials and Methods) to assay increases in theta phase coupling between the mPFC seed region and all other voxels in the brain, finding significant increases in the right anterior medial temporal lobe (aMTL) between baseline and cue periods (peak at [20; −16; −26], *Z*-score = 4.52, baseline PLV = 0.36 ± 0.0072, cue PLV = 0.38 ± 0.0061, Figs. 2a,c). Although the magnitude of this increase was modest (∼5%), the effect was highly consistent, being seen in 15/17 participants.

Next, we made use of the phase lag index (PLI; Stam et al., 2007) to address the possibility that the observed increase in mPFC-aMTL theta coupling was an artifact of volume conduction. In accordance with the observed changes in PLV, we identified a significant increase in PLI between the mPFC seed region and the right anterior medial temporal lobe between baseline and cue periods (peak at [22; −24; −22], *Z*-score = 3.48, baseline PLI = 0.29 ± 0.0086, cue PLI = 0.32 ± 0.0092, Fig. 2b). Again, the magnitude of this increase was modest (∼10%), but the effect was highly consistent, being seen in 14/17 participants. We also observed significant increases in the PLI with other nearby anterior cingulate cortex (ACC) and midline - but not lateral - PFC regions. Although PLI is insensitive to phase coupling with zero lag, the provenance of PLI increases so near to the seed region is hard to interpret with confidence, and no other regions cleared our significance threshold. Additional control analyses indicate that the increases in theta phase coupling with aMTL were not due to changes in eye movements or oscillatory power in seed and source voxels between conditions (see Materials and Methods).

### Theta Phase–Gamma Amplitude Coupling

**FIGURE 3:**
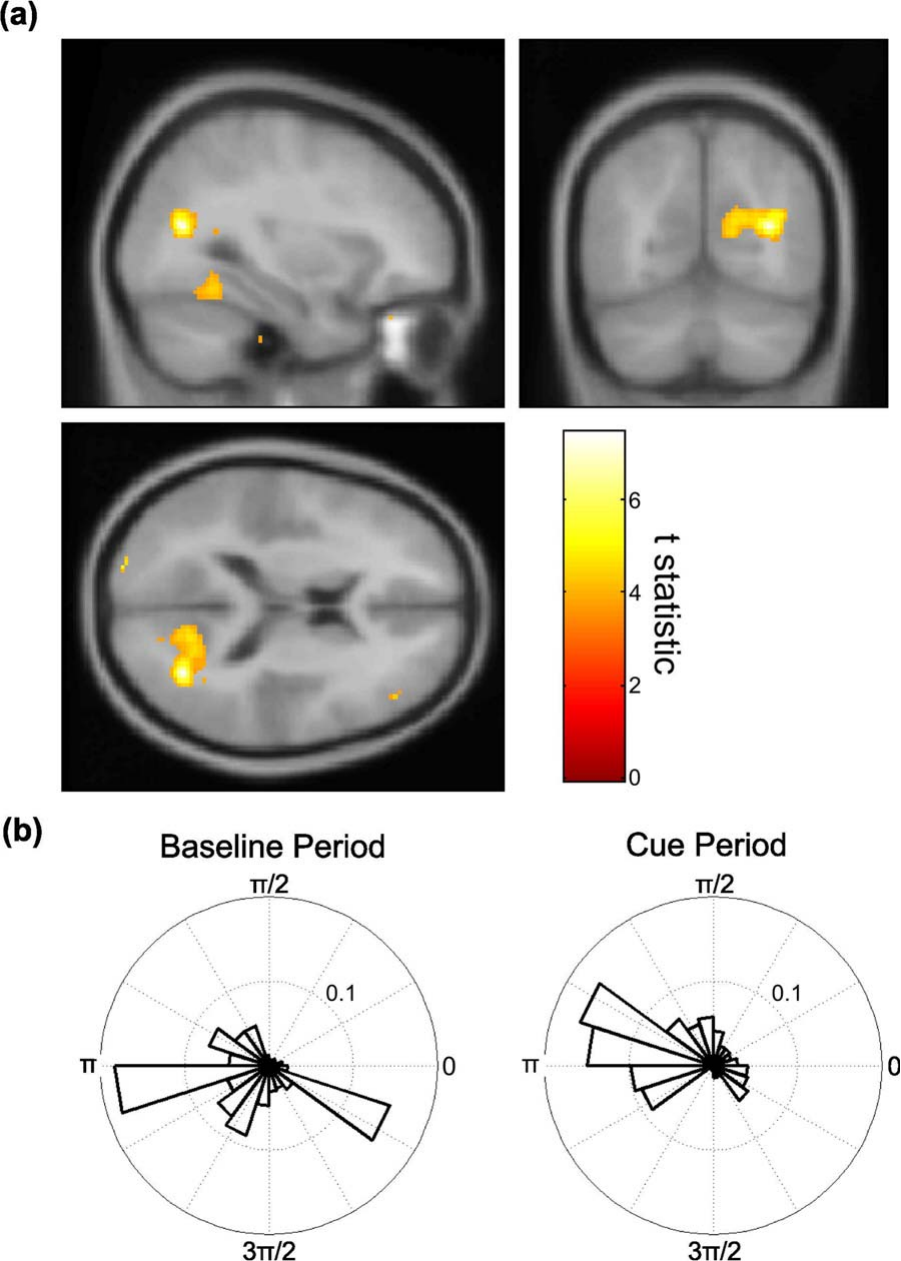
mPFC theta phase coupling with medial parietal gamma amplitude. (a) Group-averaged brain image showing a significant increase in coupling between mPFC theta phase and medial parietal cortex gamma amplitude between the baseline and cue periods, measured using the PLV (peak at [32; −66; 16], *Z*-score = 4.77). Image displayed at the statistical threshold of *P* < 0.001 uncorrected and overlaid on the canonical Montreal Neurological Institute 152 T1 image. (b) Circular histogram of phase differences between theta oscillations in mPFC seed and gamma amplitude in the group peak right medial parietal cortex voxel for a single subject at each time point during a typical baseline and cue period (baseline PLV = 0.34, cue PLV = 0.40). Note the narrower distribution of phase differences during the cue period. [Color figure can be viewed in the online issue, which is available at http://wileyonlinelibrary.com.]

Having identified an increase in mPFC-aMTL theta phase coupling between baseline and cue periods, we then used the same mPFC theta source as a seed region to explore theta phase modulation of high (65–85 Hz) gamma amplitude across the whole brain. We identified significant increases in phase-amplitude coupling between baseline and cue periods, measured using the PLV metric, in medial parietal cortex, extending into the posterior parahippocampal cortex (peak at [32; −66; 16], *Z*-score = 3.81, baseline PLV = 0.35 ± 0.0059, cue PLV = 0.38 ± 0.0059, Fig. 3). Although the magnitude of this increase was modest (∼5%), the effect was highly consistent, being seen in 14/17 participants. Moreover, control analyses indicate that this increase in theta-gamma phase- amplitude coupling was not due to changes in eye movements or oscillatory power in seed and source voxels between conditions (see Materials and Methods). No other regions cleared our significance threshold.

## DISCUSSION

We have identified an increase in mPFC theta power during the cued retrieval of learned spatial representations compared to a preceding baseline period of quiet fixation (Fig. 1b) and demonstrated increased coupling between the phase of this theta signal and both the phase of ongoing theta oscillations in the right aMTL (Fig. 2) and the amplitude of gamma oscillations in medial parietal cortex (Fig. 3).

Frontal midline theta power increases around the time of memory recall are frequently observed in human EEG studies (Klimesch et al., 2001; Summerfield and Mangels, 2005; Griesmayr et al., 2010; Addante et al., 2011), including intracranial EEG (iEEG) recorded during virtual spatial navigation (Kahana et al., 1999). Similarly, human MEG studies have identified frontal midline theta sources localized in both anterior cingulate cortex and mPFC regions (Asada et al., 1999; Ishii et al., 1999). Further research will determine whether these theta sources are distinct, that is, whether they can be dissociated by their behavioral relevance (e.g., supporting cognitive control versus memory retrieval). Moreover, due to the inherent constraints of MEG source reconstruction techniques, it is not possible for us to ascertain whether the single mPFC theta source we observe is generated by a more complex source distribution, such as two generators either side of the midline, although it is consistent with previous human studies (Asada et al., 1999; Ishii et al., 1999).

Our observation of increased theta phase coupling between mPFC and right anterior MTL during cued spatial memory retrieval suggests a role for interregional theta coupling in mnemonic function. Interactions between these two regions are thought to support memory consolidation (for review see Frankland and Bontempi, 2005), and rodent studies have demonstrated increased mPFC-MTL theta phase coupling during spatial memory tasks (Hyman et al., 2005; Jones and Wilson, 2005; Siapas et al., 2005; Benchenane et al., 2010; see Euston et al., 2012 for review). Furthermore, human electrocorticography (ECoG) studies have found increased low frequency (4–8 Hz) phase coupling between PFC and MTL during memory recall (Anderson et al., 2010) and demonstrated that the magnitude of increases in phase coupling between these regions correlates with successful memory retrieval (Watrous et al., 2013). It is interesting to note that the increased MTL-mPFC theta phase coupling observed at decision points during rodent spatial working memory tasks has a parallel in recent observations of increased theta coupling between these regions during human decision-making tasks (Guitart-Masip et al., 2013).

There is a growing body of evidence demonstrating the ability of MEG source reconstruction to localize deep brain sources, such as the MTL theta rhythm. The ability to noninvasively measure MTL activity is further supported by recent simulation studies (Quraan et al., 2011; Mills et al., 2012) and simultaneous iEEG/MEG recordings of hippocampal oscillations (Dalal et al., 2013; Staudigl and Hanslmayr, 2013). Notably, in our data, significant increases in theta phase coupling were observed with the right, but not left MTL. The right MTL is thought to be important for spatial memory (Abrahams et al., 1997; Bohbot et al., 1998; Spiers et al., 2001), while the left MTL is more commonly associated with verbal memory (Frisk and Milner, 1990). We cannot emphasize the laterality of our MTL theta coupling findings, however, as with all neuroimaging studies it is difficult to confirm whether this arises from the group statistics, and the inherent constraints of our source reconstruction technique allow for the possibility that a correlated theta source in the contralateral hemisphere could be suppressed (Barnes et al., 2003).

Our observation of increased gamma power in the visual cortex during cue presentation is consistent with a large body of experimental work suggesting that gamma power in sensory cortices is increased during perception (Gray and Singer, 1989; Tallon-Baudry and Bertrand, 1999; Hall et al., 2005; Hoogenboom et al., 2006). We also observed increased gamma power in the medial parietal cortex, an area implicated in memory retrieval (Wagner et al., 2007), spatial memory (Epstein, 2008) and imagery (Fletcher et al., 1996; Byrne et al., 2007), consistent with previous findings relating high frequency gamma (Morgan et al., 2011; Foster et al., 2012) and theta oscillations (Foster et al., 2013) in the parietal midline to mnemonic retrieval. Furthermore, we have demonstrated that gamma amplitude in this area is modulated by mPFC theta phase. Local computations in the cortex are thought to be related to the presence of gamma oscillations (see Fell et al., 2001; and reviews by Hermann et al., 2004; Jensen et al., 2007) which could be modulated by the phase of lower frequency oscillations such as theta (Buzsaki and Chrobak, 1995; Canolty et al., 2006; Lisman and Buzsaki, 2008; Schroeder and Lakatos, 2009; Sauseng et al., 2010; Jutras and Buffalo, 2010; Fell and Axmacher, 2011; Maris et al., 2011). Recent MEG and iEEG studies have implicated local MTL theta-gamma coupling in the maintenance of working memory (Axmacher et al., 2010; Fuentemilla et al., 2010; Poch et al., 2011) and contextual memory retrieval (Staudigl and Hanslmayr, 2013). Our findings suggest that human mPFC theta temporally coordinates both aMTL theta and neocortical gamma during spatial memory retrieval, although further work is necessary to determine what aspects of this task (e.g. visual cues, mental imagery, spatial memory, or general memory retrieval processes) cause these effects.

## CONCLUSIONS

Our findings suggest that theta oscillations in mPFC could work in tandem with theta in the aMTL to coordinate incoming perceptual representations from medial parietal and visual areas during cued spatial memory retrieval. Our results allow for the possibility that human frontal midline theta generated during memory retrieval interact with theta rhythms in the medial temporal lobe, and adds further support to the hypothesis that coupling of theta rhythms with neocortical gamma oscillations organizes the recruitment of information from distant neocortical sites (Buzsaki and Chrobak, 1995; Sirota et al., 2008; Buzsaki and Wang, 2012).
